# Individual predictors of sensorimotor adaptability

**DOI:** 10.3389/fnsys.2015.00100

**Published:** 2015-07-06

**Authors:** Rachael D. Seidler, Ajitkumar P. Mulavara, Jacob J. Bloomberg, Brian T. Peters

**Affiliations:** ^1^Psychology, Kinesiology, Neuroscience, Neuromotor Behavior Laboratory, University of MichiganAnn Arbor, MI, USA; ^2^Universities Space Research AssociationHouston, TX, USA; ^3^NASA Johnson Space CenterHouston, TX, USA; ^4^Wyle Science, Technology and Engineering GroupHouston, TX, USA

**Keywords:** adaptation, motor learning, predictors, genetics, motor cortex

## Abstract

There are large individual variations in strategies and rates of sensorimotor adaptation to spaceflight. This is seen in both the magnitude of performance disruptions when crewmembers are first exposed to microgravity, and in the rate of re-adaptation when they return to Earth’s gravitational environment. Understanding the sources of this variation can lead to a better understanding of the processes underlying adaptation, as well as provide insight into potential routes for facilitating performance of “slow adapters”. Here we review the literature on brain, behavioral, and genetic predictors of motor learning, recovery of motor function following neural insult, and sensorimotor adaptation. For example, recent studies have identified specific genetic polymorphisms that are associated with faster adaptation on manual joystick tasks and faster recovery of function following a stroke. Moreover, the extent of recruitment of specific brain regions during learning and adaptation has been shown to be predictive of the magnitude of subsequent learning. We close with suggestions for forward work aimed at identifying predictors of spaceflight adaptation success. Identification of “slow adapters” prior to spaceflight exposure would allow for more targeted preflight training and/or provision of booster training and adaptation adjuncts during spaceflight.

## Introduction

Personalized medicine has become a hot topic for research; many brain stimulation, physical therapy, and pharmacological approaches to movement disorders are efficacious for some individuals but not others. The ability to predict ahead of time which patients would be most responsive to differing types of treatments would clearly save time and costs, and increase patients’ quality of life. Advances have been made in identifying brain (Milot et al., 2014) and genetic (Pearson-Fuhrhop and Cramer, 2010; Kwak et al., 2013) markers of which patients will respond best to different treatments.

An interesting parallel is seen with individuals adapting movement control to the microgravity environment of space; there are large inter-individual differences in both the degree of sensorimotor disruption occurring upon initial exposure and in the recovery rate of sensorimotor control upon return to Earth. Oftentimes these individual differences are viewed as noise, i.e., measurement error; however numerous studies have exploited individual variations to better understand the neurocognitive determinants of behavior (cf. Kanai and Rees, 2011). Understanding the sources of inter-individual variability would also allow for the generation of customized pre-training and rehabilitation approaches, maximizing crewmember productivity and functional abilities. The capacity to rapidly adapt to changing gravitational environments is increasing in importance as NASA targets having the capability to send humans to Mars in the 2030s (U.S. National Space Policy, 2010).

Movement in an altered gravity environment, such as weightlessness without a stable gravity reference, results in new patterns among sensory inputs. The semicircular canals, vision and neck proprioception provide information about head tilt on orbit but without the normal otolith head-tilt position that is omnipresent on Earth. Moreover, the limb unloading that occurs means that limb “neutral” postures change and the dynamics of movement are altered. Over a few days crew members adapt to these conditions. When crew members subsequently return to Earth and are re-exposed to Earth’s 1-g environment (Earth G) the responses that are adaptive in space cause immediate maladaptive motor behavior on Earth.

### Effect of Space Flight on Upper Limb Sensorimotor Function

A number of studies of eye-hand coordination have been performed during space flight missions. The following subsections summarize some of the key evidence for eye-hand control performance decrements associated with space flight.

#### Control of Aimed Arm Movements

When astronauts first encounter an altered gravity environment, arm movements are often inappropriate and inaccurate (Johnson et al., 1975; Gazenko et al., 1981; Nicogossian et al., 1989). During the Neurolab Space Shuttle mission (STS-90), Bock et al. (2003) performed an experiment in which subjects pointed, without seeing their hands, to targets located at fixed distances but varying directions from a common starting point. Using a video-based technique to measure finger position they found that the mean response amplitude was not significantly changed during flight, but that movement variability, reaction time, and duration were all significantly increased. After landing, they found a significant increase in mean response amplitude during the first postflight session, but no change in variability or timing compared with preflight values. In separate experiments, Watt (Watt et al., 1985; Watt, 1997) reported reduced accuracy during space flight when subjects pointed to memorized targets. This effect was much greater when the hand could not be seen before each pointing trial. When subjects pointed at memorized locations with eyes closed, the variability of their responses was substantially higher during space flight than during sessions on Earth. In other studies (Berger et al., 1997; Papaxanthis et al., 1998), the investigators found that when crewmembers on the Mir station pointed to targets with eyes open, variability and mean response amplitude remained normal, but the movement duration increased by 10–20% over the course of the mission (flight day 2–162).

#### Reaching and Grasping

Thornton and Rummel (1977) showed that basic tasks such as reaching and grasping were significantly impaired during the Skylab missions. Later, Bock et al. (Bock et al., 1992, 1996a,b; Bock, 1996; Bock and Cheung, 1998) investigated pointing, grasping, and isometric responses during brief episodes of changed gravity, produced by parabolic flights or centrifugation. These experiments provided converging evidence suggesting that during either reduced or increased gravity, the mean amplitude of responses is larger than in normal gravity, while response variability and duration remains unchanged. During the Neurolab Space Shuttle mission, Bock et al. (2003) found that the accuracy during flight of grasping luminous discs between the thumb and index fingers was unchanged from preflight values, but task performance was slower.

#### Manual Tracking

Changes in the ability of crewmembers to move their arms along prescribed trajectories have also been studied in space. For example, Gurfinkel et al. (1993) found no differences in orientation or overall shape when crew members drew imagined ellipses oriented parallel or perpendicular to their long body axes with their eyes closed. In another study, Lipshits et al. (1993) examined the ability of crewmembers to maintain a cursor in a stationary position in the presence of external disturbances. They found no performance decrements when the disturbances were easily predictable. However, in a follow-on experiment using more complex disturbances, Manzey et al. (1995, 1998) found that tracking errors were increased early in flight, but gradually normalized within 2–3 weeks of exposure to the space environment. Later, Sangals et al. (1999) reported a series of step-tracking experiments conducted before, during, and after a 3-week space flight mission. Accuracy was affected only marginally during and after flight. However, kinematic analyses revealed a considerable change in the underlying movement dynamics: too-small force and, thus, too-low velocity in the first part of the movement was mainly compensated by lengthening the deceleration phase of the primary movement, so that accuracy was regained at its end. They interpreted these observations as indicating an underestimation of limb mass during flight. No reversals of the in-flight changes (negative aftereffects) were found after flight. Instead, there was a general slowing down, which could have been due to postflight physical exhaustion.

#### Force Discrimination and Control

During a MIR station mission the ability of a cosmonaut to reproduce several positions of a handle from memory was tested. The accuracy with which the handle was set to a given position was reduced; however, the temporal parameters of the movement and the number of discernable handle positions did not change (Lipshits et al., 1993; Reschke et al., 1996).

#### Fine Motor Control

Campbell et al. (2005) evaluated the feasibility of survival surgery performed on rats during the Neurolab Shuttle mission. Craniotomy, leg dissection, thoracotomy, laminectomy, and laparotomy were performed as a part of physiological investigations. Surgical techniques successfully performed on rats during space flight include general anesthesia, wound closure and healing, hemostasis, control of surgical fluids, operator restraint, and control of surgical instruments. Although the crew noted no decrement in manual dexterity, the operative times were longer compared with on Earth due to the need to maintain restraint of surgical supplies and instruments. In another study, Rafiq et al. (2006) measured the effect of microgravity on fine motor skills by investigating basic surgical task performance during parabolic flight. They found that forces applied to the laparoscopic tool handles during knot tying were increased while knot quality was decreased during flight compared with ground control sessions. Also, Panait et al. (2006) studied the performance of basic laparoscopic skills (clip application, grasping, cutting, and suturing) during parabolic microgravity flights. They found that there was a significant increase in tissue injury and task erosion and a decreased trend in the number of tasks successfully completed.

#### Dual Tasking and Manual Performance

Manzey et al. (1995, 1998) investigated motor skills in space under dual-task conditions in single case studies. They found interference between a compensatory tracking task and a concurrent memory search task to be greater in space than on Earth. The elevated interference was greatest early in flight, but gradually normalized, reaching the preflight baseline only after about 9 months in orbit. In one of these studies, Manzey et al. (1995) also found that task interference was independent of the difficulty of the memory search task, suggesting that the critical resources affected were probably not those related to memory, but rather those pertinent to motor control (both tasks required an immediate motor response). Bock and colleagues have also shown that motor-cognitive dual tasking costs are higher during microgravity portions of parabolic flight (Bock et al., 2003) and when crewmembers are on the International Space Station (Bock et al., 2010). In the latter case, manual tracking of a target was particularly affected by concurrent performance of a rhythm production reaction time task, suggesting that complex motor planning resources are particularly affected by spaceflight.

The laboratory tasks described above might actually underestimate the impairments with microgravity as many differ from real-life scenarios. For example, it was reported that microgravity slowed aimed arm movements by 10–30% on experimental tasks, but time and motion studies of routine activities on Skylab documented up to 67% slower movements in microgravity than on Earth.

### Effect of Space Flight on Posture and Locomotion

Movements are adapted during spaceflight, but this microgravity adaptive state is subsequently inappropriate for a gravitational environment so that astronauts must spend time readapting to Earth’s gravity following their return. During this readaptation period a number of studies performed at Dr. Bloomberg’s Neuroscience motion laboratory at NASA/JSC have shown disruption in astronauts’ spatial coordination abilities during walking (cf. Glasauer et al., 1995). Additionally, short and long duration spaceflight show effects of maladaptation during walking on a treadmill upon return to Earth, evidenced as increased variability of muscle activity (Layne et al., 1997, 1998, 2001). Further, during this treadmill walking test subjects showed modified lower limb kinematics, including increased variability in the support limb kinematics at heel strike as well as the motion of the knee during the double support phase (McDonald et al., 1996; Bloomberg and Mulavara, 2003; Miller et al., 2010). Further, these subjects also demonstrated disruption in head-trunk coordination after both short and long duration space flight (Bloomberg et al., 1997; Bloomberg and Mulavara, 2003; Mulavara et al., 2012). A more recent study has reported reduction in visual acuity during walking, supporting that the outcome of this increased variability in lower limb muscle activation, limb kinematics, head-trunk, and eye-head coordination after space flight is manifested as the inability to stabilize images on the retina during dynamic perturbations such as walking (Peters et al., 2011). From a functional perspective we have shown that these postflight changes contribute to impairment in mobility on a functional obstacle course (Mulavara et al., 2010) and decrement in the ability to coordinate effective landing strategies during a jump down task (Newman et al., 1997). One of the hallmark observations concerning tests of astronaut sensorimotor function is the inherent variability in both the degree of disruption and the recovery rate between subjects. Inter-subject variability in adaptive capability may account for the divergent results observed between individuals. At present we cannot predict preflight which individual astronauts will experience the most significant sensorimotor disturbances.

In the remainder of this article, we review the literature on the ability to predict the manner and degree to which each individual astronaut may be affected on sensorimotor performance after exposure to varying periods of microgravity. This would improve the effectiveness of countermeasure training programs designed to enhance sensorimotor adaptability.

While several studies have investigated adaptation to spaceflight, this work is technically difficult to conduct and therefore somewhat limited in sample sizes. Thus we also review other motor learning and sensorimotor adaptation paradigms. While sensorimotor adaptation and motor learning may not necessarily be based on the same processes as sensorimotor adaptation to spaceflight, we believe that examining this literature can lend insight into potential predictors for future study. Our goal here is to highlight factors that may be promising biomarkers of adaptability to spaceflight, providing direction for future research. We group these potential predictors based on the existing literature into categories of behavioral, brain, and genetic factors. These metrics may be biologically inter-related.

It is known that different forms of motor learning engage varying cognitive processes and neural systems (cf. Doyon et al., 2003; Krakauer and Mazzoni, 2011). While the neural bases of adaptation to spaceflight have not been explicitly examined, it is plausible that this process would be similar to visuomotor and force field adaptation tasks that have been widely studied. Sensorimotor adaptation involves a gradual updating of forward models; extensive evidence supports a role for cerebellar thalamocortical pathways in this process (cf. Imamizu et al., 2000, 2003; Tseng et al., 2007; Galea et al., 2011). A single subject case study has recently reported alterations in cerebellar-motor connectivity following approximately 5 months of spaceflight (Demertzi et al., 2015), supporting parallel cognitive and neuroplastic effects for spaceflight and model sensorimotor adaptation tasks such as adaptation to a perturbation of visual feedback. Thus, examining individual differences in sensorimotor adaptation in Earth based experiments is an important first step towards identifying predictors of spaceflight adaptability.

### Behavioral Predictors

#### Sensory Bias

We and others have shown that subjects who rely more on vision for control of movement have more difficulty adapting their walking and postural control strategies in new environments, indicating that visual dependency may predict decreased ability to adapt to novel environments (Hodgson et al., 2010; Brady et al., 2012; Eikema et al., 2013). Interestingly, Lex et al. (2012) found that individual differences in cognitive representations of movement direction were associated with rate of adaptation on a visuomotor adaptation task. Participants were asked whether pairs of movement directions were similar or not. Those that made judgments reflecting a global cognitive representation of movement directions that was aligned to cardinal axes were faster adapters. In contrast, subjects that made judgments reflecting local cognitive representations that were aligned to neighboring directions were slower adapters. Although not discussed in the article, the latter representation strategy would be more visually based and global representations would be linked to gravitational cues.

Computerized dynamic posturography (CDP) tests, which can determine the extent to which an individual effectively uses vestibular, visual, or proprioceptive cues for balance, may also serve as predictive markers of adaptation to spaceflight. Postural ataxia following space flight reflects the adaptive changes in spatial processing of sensorimotor control and the unloading effects of microgravity (Wood et al., 2011a). CDP has been used to examine postural ataxia following both short- and long duration flights, using the Sensory Organization Tests (SOTs) provided by the EquiTest System platform (NeuroCom International, 2003). The greatest decrements following spaceflight are observed when the subject’s eyes are closed and the support surface rotates in direct proportion to anterior-posterior body sway, disrupting somatosensory feedback, a protocol known as sway referencing (Paloski et al., 1992, 1994; Paloski, 1998). This condition is thus sensitive to adaptive changes in how vestibular feedback is utilized for postural control (SOT5). Most of the crewmembers had increased reliance on feedback from vision during their recovery process as a result of degraded performance of the other two feedback systems during adaptation to microgravity (Reschke et al., 1998). It has been recently demonstrated that the diagnostic performance of this test was enhanced with the addition of dynamic pitch head tilts. This finding is consistent with crewmember reports that activities of daily living requiring head tilts are more challenging during post-flight recovery, presumably due to adaptive changes in the multisensory integration of the spatial vertical (Wood et al., 2011b).

In sum, an individual’s innate sensory weighting, or the rigidity with which they adhere to a particular sensory weighting, may predict their adaptability to microgravity and subsequent readaptation upon return to Earth.

#### Behavioral Measures of Individual Motor Learning Responses as Predictors of Adaptability

Several studies have examined the time course of motor learning in different training paradigms such as a visual discrimination task (Karni and Sagi, 1993; Karni and Bertini, 1997) or while learning to adapt to either visual (Redding and Wallace, 1996) or mechanical distortions (Shadmehr and Mussa-Ivaldi, 1994; Kording et al., 2007). The time course of motor learning occurs in two stages: (1) a fast, within-session improvement that can be induced by a limited number of trials on a time scale of minutes; and (2) a slowly evolving, incremental performance gain, triggered by practice but taking hours to become effective (Karni and Bertini, 1997). Two distinct neural systems that differ from each other in their sensitivity to error and their rates of retention have been identified (Smith et al., 2006). Separate neural substrates may control the execution of these two motor strategies (Pisella et al., 2004; Luauté et al., 2009). Pisella et al. (2004) reported that a patient with a bilateral lesion of the posterior parietal cortex (PPC) was not able to implement on-line strategic adjustments in response to a prismatic shift in visual feedback during a pointing task, yet showed adaptive after-effects, suggesting that the strategic component was linked to the PPC, and the adaptive component was linked to the cerebellum. Anguera et al. (2010) showed that cognitive processes such as spatial working memory contributed to the early and not the late stage of sensorimotor adaptation by comparing the rates of adaptation and overlap of the neural substrates underlying these two motor learning stages during a visuomotor adaptation task. Strategic motor control occurs early in the adaptation process once the subject becomes aware of the sensory manipulation and understands on some conscious level how to correct for it (Redding and Wallace, 1996; McNay and Willingham, 1998; Seidler, 2004). For example, subjects exposed to a prismatic lateral shift in vision make strategic corrections in pointing movements based on visual feedback to improve performance and eventually point directly to a target (Weiner et al., 1983; Rossetti et al., 1993; Welch et al., 1993). Bock and Girgenrath (2006) investigated the strategic and adaptive realignment components of sensorimotor adaptation of arm aiming movements in response to distorted visual feedback in young and older adults. They found that the recalibration processes were not impaired in older adults compared to young adults, as shown by the magnitude of after effects and transfer of adaptation to novel sensorimotor arrangements, but the strategic processes as represented by improvements during exposure were degraded (Bock, 2005; Bock and Girgenrath, 2006).

These findings support the notion that individual capability for strategic and plastic-adaptive responses shown in behavioral adaptability tests may predict the rate of adaptation to microgravity and re-adaptation upon return to Earth.

#### Cognitive Factors

Visuomotor adaptation involves the recalibration of a well-learned spatial-motor association. There is evidence to support that visuomotor adaptation is cognitively demanding, at least in the early stages (Eversheim and Bock, 2001; Taylor and Thoroughman, 2007, 2008). For example, Eversheim and Bock (2001) used dual task paradigms to demonstrate that cognitive resources are engaged in a time-dependent fashion during adaptation: resources related to spatial transformations and attention were highest in demand early in adaptation, while those related to movement preparation were more in demand later in learning.

We have investigated whether individual differences in spatial working memory capacity relate to the speed of adaptive performance changes in a visuomotor adaptation paradigm (Anguera et al., 2010). Variation exists in the number of items that individuals can hold and operate upon in working memory (cf. Vogel and Machizawa, 2004), making it particularly amenable to individual differences research approaches. For example, individual differences in working memory capacity have been found to be predictive of math problem solving (Beilock and Carr, 2005). We investigated the contribution of working memory and other cognitive processes to sensorimotor adaptation by administering a battery of neuropsychological assessments which measured abilities in attention, processing speed, verbal and spatial working memory. Participants also adapted manual aiming movements to a 30° clockwise rotation of the visual feedback display about the central start location. We divided the learning curve into “early” and “late” components for each individual. We found that performance on the card rotation task (Ekstrome et al., 1976), a measure of spatial working memory, was correlated with the rate of early, but not late, learning on the visuomotor adaptation task. Importantly, there were no correlations between measures of verbal working memory and either early or late learning, suggesting that spatial working memory capacity is a specific predictor of early visuomotor adaptation rate.

These findings support the notion that individual differences in spatial working memory capacity may also serve as an effective predictor of spaceflight sensorimotor adaptation success.

#### Motor Variability

Wong and Shelhamer (2014) reported that the rate at which participants adapt saccadic eye movements in response to a double-step target displacement (target location changes during a saccade) was predicted by the extent to which baseline saccade errors were correlated across trials. This suggests that common error detection and correction mechanisms may be at play both in normal motor control and during adaptive modifications to behavior. In a learning to learn paradigm where subjects progressively adapted pointing movements to several different visual distortions across test sessions, we have reported that participants increase their adaptability; that is, they adapted to each subsequent perturbation faster than control subjects (Seidler, 2004). We found that these subjects were more affected by transient, unlearnable perturbations than controls, however, suggesting that learning to learn may be associated with less stable baseline performance. That is, a strong sensitivity to errors may be beneficial to learning, as long as the environment is consistent and predictable. When environmental changes are transient however, this sensitivity to motor errors would be disadvantageous, potentially resulting in behavioral modifications toward inappropriate goals.

In a recent study, Wu et al. (2014) also reported that faster sensorimotor adaptation is associated with greater baseline variability, particularly in task relevant dimensions. For example, baseline variability related to velocity was predictive of adaptation to a velocity-dependent force field perturbation applied to arm movements. In sum, these studies suggest that baseline measures of motor variability may be useful predictors of subsequent adaptability.

### Neural Predictors

#### Brain Activity

As part of our efforts to investigate cognitive predictors of sensorimotor adaptability described above (Anguera et al., 2010, 2011), we had participants perform a manual sensorimotor adaptation task and a spatial working memory task in an MRI scanner. We found that the neural correlates of early adaptation overlapped with neural substrates that participants engaged when performing a spatial working memory task, notably in the right dorsolateral prefrontal cortex and in the bilateral inferior parietal lobules. There was no neural overlap between late learning and spatial working memory. We also tested a group of older adult participants for comparison (Anguera et al., 2011), and found that across the young and older adults, the extent to which participants recruited the right dorsolateral prefrontal cortex explained individual differences in the rate of early adaptation. That is, the more that participants recruited this brain region, known to be involved in spatial working memory processes, the faster they progressed through the early, strategic stage of sensorimotor adaptation regardless of their age. Engagement of this brain region may be correlated with spaceflight sensorimotor adaptation.

A study by Burke et al. (2014) investigated a number of potential clinical and neural predictors of recovery of lower limb function in patients that have experienced a stroke. They found that the leg Fugl-Meyer score—a clinical scale of function—and the extent of motor cortex that was recruited during movements of the ipsilateral foot (assessed with functional MRI) provided the best predictors of subsequent treatment gains (Burke et al., 2014). These findings support the notion that brain activity can provide a useful prediction of future learning/sensorimotor adaptability.

#### Brain Connectivity

Recent approaches in MRI have been developed to assess brain *functional (resting state functional connectivity, fcMRI)* and *structural (diffusion weighted MRI, DTI)* network connectivity, allowing for more integrative assessments of distributed neural systems than in the past. Data acquisition for both techniques is rapid and non-invasive. Moreover, because participants are not performing a task, there are no confounds of the effects of fatigue, attention, or task difficulty that often complicate interpretation of task-driven fMRI studies. FcMRI has proven fruitful for the study of large-scale brain networks in healthy and diseased individuals. Low frequency blood oxygen level dependent (BOLD) signal fluctuations in remote but functionally related brain regions show strong correlations during the resting state (Biswal et al., 1995). These correlations are highly spatially structured, following known anatomical networks, and are therefore thought to reflect functional connectivity of the human brain. Functional networks that have been identified with fcMRI in healthy individuals by our group and others include motor cortical networks (Biswal et al., 1995; Peltier et al., 2005), striatal thalamo-cortical networks (Kelly et al., 2009), and cerebellar thalamocortical networks (Krienen and Buckner, 2009; Bernard et al., 2012, 2014).

Resting state network correlations have behavioral relevance. For example, default mode network connectivity is altered following visual (Lewis et al., 2009) or motor (Albert et al., 2009) learning. Furthermore, we have shown that the magnitude of corticostriatal network connectivity in Parkinson’s patients is correlated with their motor (putamen networks) and cognitive (caudate networks) symptoms, and is modulated by dopaminergic medication (Kwak et al., 2010). Moreover, we have documented that greater resting state motor interhemispheric connectivity in older adults is correlated with “motor overflow”, or recruitment of the ipsilateral motor cortex during a unimanual task (Langan et al., 2010). Similarly, DTI metrics of white matter tract integrity exhibit network-selective correlations with behavior (Della-Maggiore et al., 2009). For example, white matter integrity underlying the left, but not the right, Broca’s area is correlated with the ability to learn an artificial grammar (Flöel et al., 2009), while cerebellar white matter fractional anisotropy is correlated with individual differences in the rate of motor learning (Della-Maggiore et al., 2009; Tomassini et al., 2011).

The cerebellum plays a critical role in sensorimotor adaptation (Martin et al., 1996a,b). We have shown that individual differences in regional cerebellar lobule volumes are predictive of balance scores (Bernard and Seidler, 2013), and cerebellar network functional connectivity is predictive of sensorimotor and cognitive function in older adults (Bernard et al., 2013). Studies in patients with cerebellar damage following stroke support involvement of Crus I and lobule V in adaptive improvements with practice, whereas lobules V and VI are linked to aftereffects and retention of adaptation (Werner et al., 2010).

Another resting state measure of neural function is corticospinal excitability, measured as the magnitude of a motor evoked potential (MEP) elicited with a transcranial magnetic stimulation (TMS) pulse applied to motor cortex. Interestingly, stroke patients with a lower baseline MEP benefited more from subsequent robotic training (Milot et al., 2014). These results, combined with the preceding discussion, support the notion that resting state measures of neural connectivity, excitability, and pathway integrity are predictors of future skill learning. A more thorough elucidation of the neural substrates of microgravity sensorimotor adaptation may yield similar predictive brain network metrics.

### Genetic Predictors

Genetic polymorphisms have been shown to be associated with factors including neuroanatomical phenotypes such as cortical size or integrity of gray and white matter in the brain (Rimol et al., 2010) and neuroplasticity (Pearson-Fuhrhop and Cramer, 2010). Recent findings of genotype associations with dopamine availability in the prefrontal cortex and corticostriatal circuits highlight the role of a single nucleotide polymorphism of the catechole-O-methyltranspherase (COMT) gene at codon 158/108 (Frank et al., 2009). The substitution of a Valine (*val*) with Methionine (*met*) allele at this codon (G to A) results in reduced COMT enzymatic activity, which leads to less dopamine degradation and higher prefrontal dopamine availability (Chen et al., 2004). COMT *met* homozygotes show comparatively better performance in working memory tasks and other measures of executive function (cf. Malhotra et al., 2002). In addition to COMT *val158met*, the DRD2 *G > T* polymorphism influences dopamine availability by regulating the expression of striatal dopamine receptors. D2 receptor activity in the striatum has been associated with motor control, coordination, and error avoidance (Xu et al., 2007; Doll et al., 2011). The *T* allele of the DRD2 genotype (rs 1076560) is associated with reduced D2 expression and consequently with declines in cognitive and motor processing (Bertolino et al., 2009). Individuals who are carriers for the DRD2 *T* allele show a greater area of activated brain regions and reduced levels of performance in working memory tasks, indicating less efficient neural processing (Zhang et al., 2007).

Another candidate gene that may be an effective predictor of sensorimotor adaptability is brain-derived neurotrophic factor (BDNF), which is associated with brain derived neurotrophic factor, an important modulator of brain plasticity and learning. BDNF *val/met* carriers have reduced BDNF in comparison to *val/val* and have recently been demonstrated to exhibit reduced manual sensorimotor adaptation and less retention of adaptive learning (Joundi et al., 2012). Finley et al. (2004) have also reported that variation in the α2-adrenergic receptor genetic polymorphism is associated with individual differences in autonomic responses to stress, including susceptibility to motion sickness, an important factor influencing functional performance and productivity in spaceflight that has been linked to vestibular system functioning (Oman and Cullen, 2014).

We evaluated whether a particular DRD2 polymorphism (rs 1076560, G > T), which codes for D2 dopamine receptors in the striatum, is associated with how well patients with Parkinson’s disease respond to exogenous administration of dopamine via L-DOPA (Kwak et al., 2012). DRD2 *T* allele carriers of this polymorphism have reduced D2S expression (short isoform of the D2 receptor); thus in comparison G allele carriers have higher D2 receptor availability. Our hypothesis that Parkinson’s patients who are minor *T* allele carriers would exhibit a greater benefit of levodopa on early stage motor sequence learning was tested in a behavioral study with 45 Parkinson’s patients. Patients were tested on two days following a single blind placebo controlled design, with administration of levodopa or placebo pill in a counterbalanced fashion across the two test days. Levodopa improved sequence learning over the level of placebo pill for only the TT and GT patients, whereas GG patients did not show a benefit from levodopa (Kwak et al., 2012). These findings support the notion that treatment plans for patients with Parkinson’s disease can be enhanced by taking into account measures of endogenous dopamine availability such as genotype. Similar findings were observed in a study of healthy participants by Pearson-Fuhrhop et al. (2013); individuals with a greater number of beneficial alleles for genetic polymorphisms involved in dopaminergic metabolism exhibit greater motor skill learning gains. Meanwhile, only participants with a lower number of beneficial alleles exhibited improvements in motor learning with levodopa administration.

Recently we evaluated the proposed role of alleles for genes involved in dopaminergic transmission (COMT *val158met*, and DRD2 *G > T*) as an index of individual differences in motor sequence learning and visuomotor adaptation (Noohi et al., 2014). To test the hypothesis that individuals homozygous for high performance-associated alleles (COMT-*met* and DRD2-*G*) would demonstrate faster rates of motor learning and adaptation we tested 70 young adult females. The minor allele groups (*val-val* for COMT and *TT* for DRD2) exhibited overall slower reaction time on the motor sequence learning task for both random and sequence blocks, indicating poorer performance but no difference in learning. Of particular interest, we found COMT *val-val* individuals adapted manual aiming movements to a visual distortion more slowly than *met-met* or *val-met*. We also combined COMT and DRD2 polymorphisms into a single model by quantifying the number of “high performance” alleles that each individual carries, and by using non-parametric linear regression we found an association between the rate of sensorimotor adaptation and number of high performance alleles. Thus, one’s genotype for genes involved in dopaminergic metabolism may also serve as a predictor of spaceflight adaptability.

## Conclusions and Recommendations for Forward Work

There are not any proven predictive biomarkers of human spaceflight adaptability available at this time. However, the literatures on predictors of motor learning, sensorimotor adaptation, and of functional recovery following stroke suggest several that would be fruitful for future investigation. For example, the BDNF polymorphism and other genes that are involved in dopaminergic metabolism have been linked to manual sensorimotor adaptability, as outlined above. Promising next steps for forward work would include examining whether these polymorphisms are associated with individual differences in locomotor adaptation tasks that have been shown to be sensitive indices of spaceflight adaptation effects (cf. Mulavara et al., 2010), or whether they associate with adaptation to spaceflight analog environments such as long duration head down tilt bed rest. Furthermore, one could examine retrospective data on spaceflight adaptation and aftereffects and link them to crewmembers’ genetic data.

Another productive approach would include pretesting future crewmembers using a number of the brain and behavioral approaches outlined in this review. For example, multiple tests of relative reliance on visual vs. vestibular inputs have been validated in the literature. It would also be important to understand how these sensory biases interact with sensorimotor adaptation processes; this could be studied in ground-based experiments. We hypothesize that greater reliance on one sensory input would hinder detection of multisensory conflict, thereby slowing adaptive processes. Thus it may be that a greater reliance on visual vs. vestibular inputs would decrease spaceflight-induced orientation conflict, but it could also slow re-adaptation of sensorimotor control upon return to Earth.

Given that sensorimotor adaptation is known to rely upon cerebellar circuitry (Werner et al., 2010; Galea et al., 2011), regional cerebellar volumes and cerebellar-cortical connectivity strength may also serve as predictors of spaceflight adaptation success. It will be critical to understand how these metrics relate to variations in sensorimotor adaptation rates in ground based experiments and spaceflight analog environments before preflight testing in crewmembers.

Identifying individual predictors of spaceflight adaptability can lead to personalized pre-flight adaptive training, as well as inflight booster training, which could be coupled with technological and/or pharmacological adjuncts for those predicted to have difficulty with sensorimotor adaptation. The success of identifying predictive biomarkers will hinge significantly upon large sample sizes and predictive statistics to identify critical cut points for “faster” vs. “slower” adapters.

## Conflict of Interest Statement

The authors declare that the research was conducted in the absence of any commercial or financial relationships that could be construed as a potential conflict of interest.
